# Developing an Algorithm to Quantify Dermatomyositis-Associated Calcinosis Using Infrared Images

**DOI:** 10.3390/diagnostics16213600

**Published:** 2026-11-01

**Authors:** Saahil Sachdeva, Briana Cervantes, Marcus Y. Chen, Lisa G. Rider, Adam Schiffenbauer

**Affiliations:** 1 National Institute of Bioengineering and Biomedical Imaging, Bethesda, MD 20892, USA; 2 Environmental Autoimmunity Group, National Institute of Environmental Health Sciences, Bethesda, MD 20892, USAriderl@mail.nih.gov (L.G.R.); 3 National Heart, Blood, and Lung Institute, Bethesda, MD 20892, USA

**Keywords:** calcinosis, thermography, imaging algorithm, DM, myositis

## Abstract

**Background/Objectives**: Adult and Juvenile Dermatomyositis (DM/JDM)-associated calcinosis varies in its clinical presentations. Current modalities such as physician exam and CT imaging do not fully document calcinosis burden. As a result, this paper examines infrared imaging, which measures skin temperature, as a supplementary modality to assess calcinosis activity. **Methods**: Fifteen patients (3 DM and 12 JDM) who fulfilled probable or definite Bohan and Peter criteria for DM/JDM and with previously documented calcinosis were included. IR scans of target body regions were obtained in a temperature-controlled and humidity-monitored room. We developed an algorithm to assess calcinosis by measuring local temperature variations in IR scans. Body regions were determined to be calcinosis-positive if the algorithm identified at least 15% of the body region as suspected calcinosis. **Results**: Twenty-nine out of ninety body regions were designated calcinosis-positive. The average suspected calcinosis areas ranged from 8.6 to 14.2%, and left and right extremity averages were within 0.6% area of each other. Agreement between the IR algorithm and physician exam was variable, as the algorithm simultaneously detected regions that overlapped with physician-marked areas and regions outside of physician-marked boundaries. **Conclusions**: IR imaging appears to identify areas of calcinosis activity in DM/JDM and presents many promising advantages to warrant future study. Its integration with structural imaging modalities will enable a more comprehensive assessment of calcinosis.

## 1. Introduction

Dermatomyositis is a rare autoimmune disease characterized by chronic muscle and skin inflammation [1]. The condition can be classified as juvenile dermatomyositis (JDM) or adult dermatomyositis (DM) if the onset occurs in childhood vs. adult years.

One potential complication of DM/JDM is calcinosis, the heterotopic calcification of soft tissue that can manifest in many places in the body, with higher frequency of lesions in the trunk and distal and proximal extremities [2]. These lesions cause patients major functional disability by inducing pain, soft tissue swelling, joint contractures, and/or ulcers [3,4]. Furthermore, each lesion varies drastically in its depth, either classified as superficial or deep, and in its density, appearing hardened or liquified [5]. These heterogeneities in calcinosis can make detection difficult by physical exam.

One critical aspect of monitoring calcinosis is imaging, yet current modalities fall short of addressing the full spectrum of clinical needs. Plain radiographs, the most common modality, create 2D images with limited detection sensitivity to early calcinosis onset. Computed tomography (CT) scans produce high-resolution 3D images, but are expensive, require specialized equipment and clinical infrastructure, and expose patients to ionizing radiation. Ultrasound, though inexpensive and non-ionizing, is less reproducible and may be inaccurate in areas of extensive calcinosis. Finally, magnetic resonance imaging, used often for diagnosing idiopathic inflammatory myopathies, fails to capture calcinosis reliably, due to each lesion’s varying water content [5]. Due to these limitations, there is room for superior imaging modalities to be developed.

Infrared imaging (IR) is a light-based modality that captures an object’s emitted infrared waves to calculate its surface temperature. It has been used previously for other medical applications, including screening for COVID-19, diagnosing type II diabetes, and monitoring glaucoma as it provides temperature information unavailable through other modalities [6]. Thermography has also been demonstrated as a quantifiable tool for disease activity, such as in rheumatoid arthritis [7]. This has demonstrated the ability of infrared thermography to detect subclinical inflammation involving cell types similar to those seen in DM/JDM [8]. However, no existing studies have applied IR to DM-associated calcinosis, which is characterized by elevated macrophage activity and pro-inflammatory cytokines [9]; this inflammatory activity increases local metabolic demand and blood flow, generating heat that is expected to be detectable at the skin surface [10]. As a result, healthy controls typically have moderate skin temperatures with minimal local variations, so abnormalities may manifest through extreme temperatures and/or higher variations.

We assessed the ability of an original IR processing algorithm to assess calcinosis activity as a function of skin temperature. We believe its utility will provide clinicians with a supplementary, non-ionizing, inexpensive, and easy-to-use imaging modality.

## 2. Materials and Methods

Study subjects were recruited from a myositis natural history study conducted at the National Institutes of Health (NIH) Clinical Center in Bethesda, MD, and enrolled from August 2020 to July 2022. Inclusion criteria for the present study were probable or definite DM/JDM by Bohan and Peter criteria. Each patient also had suspicion of calcinosis, either self-reported or by documented assessment from a medical professional. Exclusion criteria included any case where the procedures were not deemed medically appropriate by the treating physician or principal investigator. Patients were not excluded based on their medication regiment.

This study was conducted in accordance with the Declaration of Helsinki, and the protocol was approved by the NIH Institutional Review Board. All patients provided written informed consent.

Patients refrained from eating, drinking, exercising, or applying makeup for four hours before scanning. For the session, patients dressed in a hospital gown. All scans were conducted by an IR camera (ImageIR^®^ 8300, InfraTec, Dresden, Germany). All scans were done in a temperature-controlled and humidity-monitored room (18–22 °C, 50–60% humidity). Target scan locations were selected in advance of the procedure, based on common calcinosis areas in DM/JDM, including the abdomen, back, arms, and legs. All images were taken as close to perpendicular to the patient as possible, and body regions were exposed and isolated with use of thermal mats.

The InfraTec ImageIR^®^ 8300 IR camera has an image resolution of 640 × 512 pixels, a pixel pitch of 15 µm, and a temperature accuracy of ±1K. Its thermal resolution is rated at 20–25 mK for an object at 30 °C. Each image was captured with a thermally decoupled standard lens with a focal length of 25 mm, F-number of 2.0, and a FOV of 384 by 307 mm at a 1 m distance for 1 s intervals over a 20 s scan. The camera was calibrated by the manufacturer before the start of the study.

All patients underwent a comprehensive clinical evaluation, including a detailed medical history and self-administered questionnaires capturing demographic and disease-specific variables (sex, race, ethnicity, date of diagnosis, and autoantibody status). Patients also received a standardized physical examination to assess the extent and sites of calcinosis.

Physician-reported outcomes include the Physician Global Calcinosis Severity assessment, a composite measure of calcinosis activity that incorporates lesion extent, burden, size, impact, and warmth, and the Physician Global Activity Assessment, which evaluates overall disease activity based on all available clinical information. These assessments were recorded along a continuous ten-centimeter visual analog scale. Patients were also evaluated by the Cutaneous Dermatomyositis Disease Area and Severity Index (CDASI), which grades skin disease activity by the severity and extent of erythema and cutaneous damage by the presence and extent of calcinosis in 15 different body areas [11].

Through IRBIS 3.0 software (InfraTec, Dresden, Germany), infrared images were extracted as .CSV temperature maps and as .TIFF images for visualization. All algorithm analysis was performed on the .CSV files to leverage the camera’s accuracy and resolution and to mitigate errors associated with direct image processing [12]. All subsequent steps are performed in MATLAB 2024a (Mathworks, Natick, MA, USA) with the Image Processing Toolbox version 2024a (Mathworks, Natick, MA, USA).

## 3. Results

### 3.1. Demographics

The study cohort included 15 patients with DM/JDM-associated calcinosis, the majority of whom were female (73%). The cohort was predominantly White (87%), with smaller representation of African Americans (20%) and Asians (7%). The mean age at imaging was 27 ± 16 years, with a mean disease duration of 13.6 ± 14.3 years. Most patients had a diagnosis of JDM (73%). On average, physician global disease activity was low (mean 1.3 ± 1.2), while calcinosis severity was moderate (mean 3.8 ± 2.1) [Table 1].

### 3.2. Use of IR as a Medical Imaging Modality

**Figure 1:**
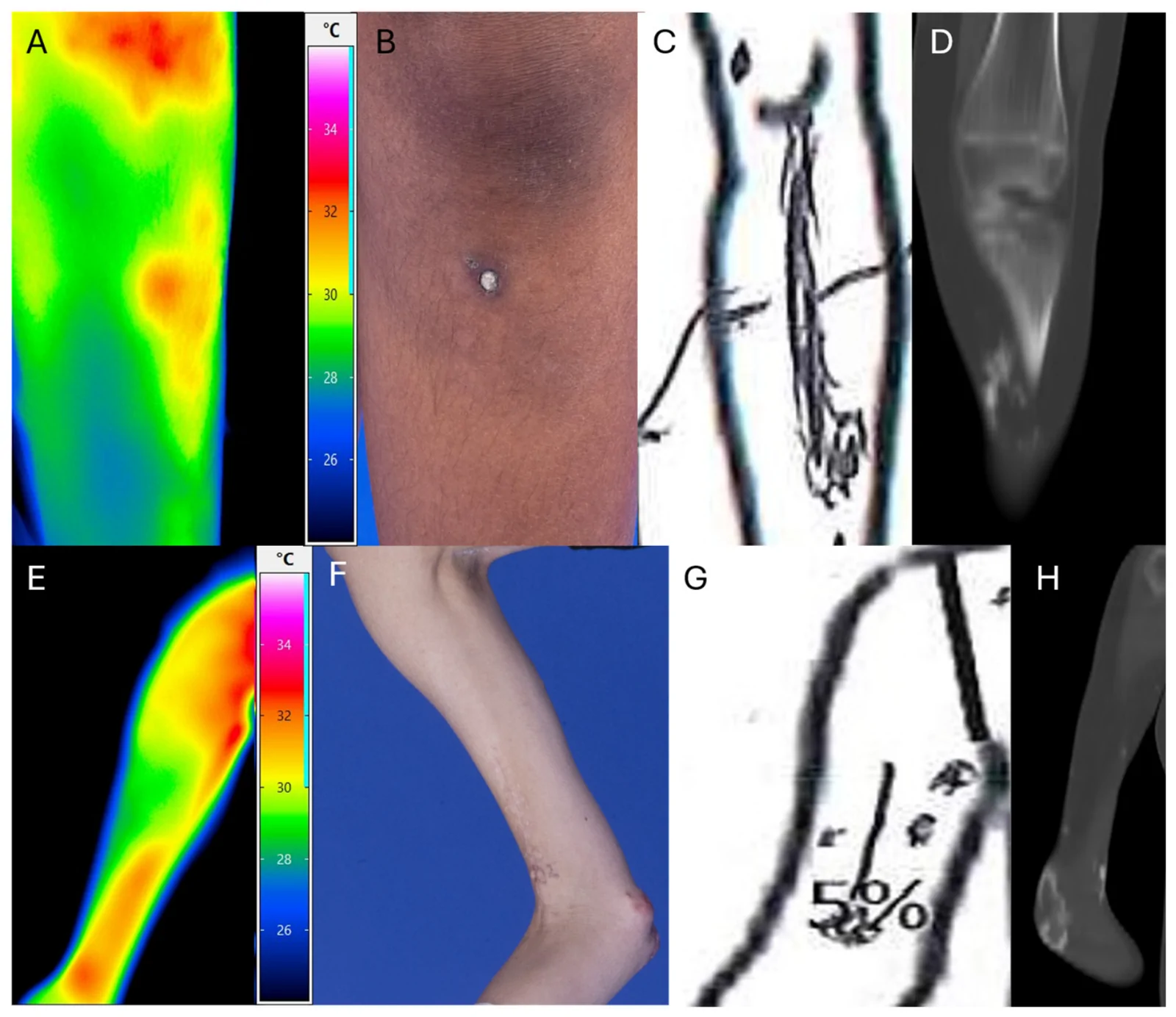
Patient 1 (Images (**A**–**D**)) had calcinosis in their left lower extremity distal to the knee. Image (**A**) is the IR image of this area and image (**B**) is the traditional photo of the same area. Image (**C**) is the physician drawing of the calcinosis from physical exam. Image (**D**) is the CT scan of the same area. Patient 2 (Images (**E**–**H**)) had calcinosis in their right upper extremity proximally to the elbow. Image (**E**) is the IR image of this area and Image (**F**) is the traditional photo of the same area. Image (**G**) is the physician drawing of calcinosis from physical exam. Image (**H**) is the CT scan of the same area. Visually, calcinosis lesions identified by physical exam and/or CT as warm appear as red/pink in IR, indicative of a higher skin temperature. Abbreviations: IR, infrared imaging; CT, computed tomography.

Two hundred and fifty-seven IR images from 3 DM and 12 JDM patients (age 9–60) were obtained from six body regions consisting of the right/left upper/lower extremities, abdomen, and back. Patients with DM-associated calcinosis often exhibited local inflammation, manifesting as areas of extreme skin temperature and areas with higher local variations [13]. All predetermined image areas were captured for all patients, there were no injuries from the procedure, and all additional areas of interest could be captured without limitations to accessing the site. This is particularly noteworthy as this population included several individuals with significant contractures and mobility issues that restricted their ability to be positioned for other testing but did not impact the IR imaging process. The hot and cold areas identified by IR present a visual indication of some underlying abnormalities found in modalities such as traditional photography, physician exam, and CT scans (Figure 1).

### 3.3. Algorithm Development

We hypothesized that regions with certain local temperature variations indicate a calcinosis inflammatory signature. As such, the proposed algorithm first sets a temperature threshold of 25 °C to eliminate the background. Next, the algorithm employs a standard deviation threshold of 0.05 to 0.1 °C within 5 × 5 sliding pixel windows to identify regions of temperature variability indicative of inflammation. Any window including background information was omitted from consideration.

The percent calcinosis area (PCA) was calculated as the number of pixels determined to contain calcinosis by the algorithm divided by the number of pixels determined to contain body area, multiplied by 100 to express the result as a percent. To account for additional inflammatory sources, the region was defined as calcinosis-positive when PCA exceeded 15% of the region’s area. This threshold was selected empirically to reduce false positives while maintaining sensitivity to true lesions.

The algorithm identified images as calcinosis-positive (PCA ≥ 15%) in 29 of 90 (32.2%) body regions imaged. Among images classified as positive, PCA values ranged from 15.0% to 29.0%. Detection frequency varied by anatomical location, with the highest rates observed in the left upper extremities (6/15 patients, 40.0%) and left lower extremities (6/15 patients, 40.0%) (Table 2). Lower detection frequencies were observed in the back (4/15 patients, 26.7%), where one image showed 0.0% PCA; the only instance of complete absence of algorithm-detected calcinosis.

Bilateral symmetry in detection patterns was apparent in several body regions. The upper extremities showed comparable detection frequencies between right and left sides (13.2% vs. 14.2%), as did the lower extremities (both 11.4% vs. 12.1%). The abdomen region exhibited the highest variability in PCA values (standard deviation 8.9%), while the right lower extremities showed the least variability (standard deviation 4.0%) (Table 2).

**Figure 2:**
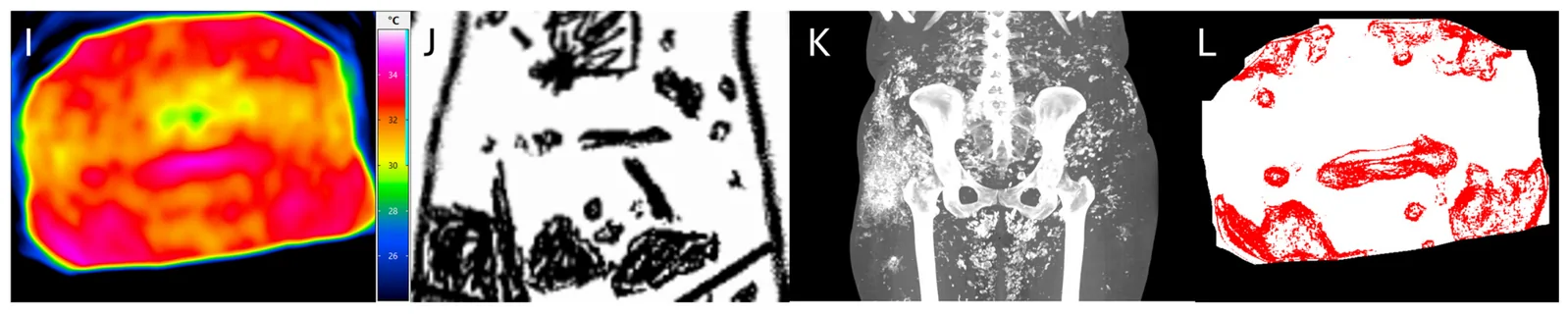
Patient 3 had calcinosis in their lower abdomen. Image (**I**) is the IR image of this area that shows abnormal patterns of temperature, as indicated in red and pink. Image (**J**) is the physician drawing of the calcinosis from physical exam. Image (**K**) presents the CT scan of the imaged region, highlighting the structural extent of calcinosis deposition. Image (**L**) is the algorithm’s output, where red indicates areas of suspected calcinosis. Abbreviations: IR, infrared imaging; CT, computed tomography.

When the algorithm identified suspected calcinosis, the positive pixels appeared in both clustered formations and as isolated detections within the same image. Both patterns contributed to the overall PCA calculation and determination of calcinosis-positive status. The algorithm predominantly highlighted regions corresponding to areas of elevated temperature on the infrared images, indicating its utility to measure calcinosis activity (Figure 2).

The algorithm also successfully identified pixels within physician-marked calcinosis regions in multiple cases. However, the agreement between algorithm detection and physician annotations was incomplete. In nearly all calcinosis-positive images, the algorithm simultaneously detected regions that overlapped with physician-marked areas, and regions outside of physician-marked boundaries. Conversely, some physician-annotated regions did not trigger algorithm detection.

## 4. Discussion

This study represents the first exploratory application of infrared imaging for detecting calcinosis in DM/JDM patients. Our novel algorithm identified temperature variations consistent with inflammatory activity in regions where calcinosis was present, suggesting that IR imaging may serve as a complementary tool for monitoring this challenging complication. The bilateral symmetry observed in several cases (Table 2) supports the potential of detecting true pathological changes rather than random artifacts, as calcinosis often manifests symmetrically in DM/JDM patients.

The inconsistencies between the physician drawing and the IR algorithm suggest that while calcinosis is associated with inflammation, the relationship between lesion characteristics and skin temperature is complex. For example, factors such as depth, tissue density, tissue thermal conductivity, perfusion rate, metabolic heat generation, and blood temperature may affect the detectability of a lesion’s thermal signature by IR [14].

Importantly, the algorithm may also detect inflammatory signatures beyond calcinosis alone. DM is associated with multiple inflammatory processes, including skin, skeletal tissue, pannicular, and myofascial inflammation, all of which could produce temperature variations captured by IR imaging. This lack of specificity represents both a limitation and a potential opportunity: while the algorithm cannot definitively distinguish calcinosis from other inflammatory processes, it may provide valuable information about overall disease activity.

This exploratory study has several important limitations that must be acknowledged. First, the sample size was small, with limited healthy control subjects for comparison. This restricts our ability to establish normative temperature variation ranges and to determine the algorithm’s specificity for pathological findings. As a result, the parameters used require refinement through systematic validation studies. Also, the edges of each body region were removed from consideration due to their high temperature variation against the image background, resulting in a false negative despite many areas of calcinosis being at the edges of the body. Furthermore, setting the calcinosis-positive threshold to 15% limits diagnostic sensitivity to areas with smaller lesions.

Despite its limitations, IR offers several advantages that warrant further investigation. The modality is non-ionizing, inexpensive, portable, and easy to use, making it accessible for frequent monitoring without the radiation exposure of CT scans or the cost and complexity of MRI. These characteristics are especially valuable for pediatric patients with JDM, as they are at higher risk of cumulative radiation exposure and may be less likely to tolerate sitting still for a prolonged MRI. This differentiator gives IR a great risk profile for longitudinal monitoring of calcinosis extent.

IR also enables serial monitoring of disease activity, a critical gap in current calcinosis assessment. Modalities such as CT and plain radiographs can detect established lesions but provide limited information about inflammatory activity and are unable to differentiate between active and inactive lesions. This capability to assess activity rapidly could be particularly valuable for evaluating the effectiveness of anti-inflammatory therapies, where changes in metabolic activities may occur before structural changes visible on conventional imaging.

From a practical standpoint, IR requires minimal specialized training and infrastructure. Unlike CT and MRI, which require dedicated imaging suites, certified technologists, and patient positioning within enclosed scanners, IR is a non-contact modality that can be performed without direct interaction with the patient. Though imaging requires a temperature-controlled room, the camera can be operated by trained clinical staff after brief formal instruction. The system’s portable nature also enables integration into routine clinical visits, supporting rapid, longitudinal assessment of calcinosis progression.

There is currently no established gold standard for the assessment of calcinosis burden and activity despite the promising capabilities of IR. Physician assessment of calcinosis lesions and mapping their locations remain the closest clinical reference, while CT scans provide crucial structural information [2]. Yet, an important open question is how to interpret discordant findings between IR, conventional imaging modalities, and physician assessment of calcinosis lesions.

Future work should therefore focus on positioning IR within a multimodal framework for calcinosis evaluation rather than as a standalone tool. This includes strict correlation studies of the IR algorithm’s output to CT imaging and physical exam, as well as studies improving specificity by distinguishing calcinosis-related inflammation from other coexisting inflammatory processes. Additionally, investigating the relationship between lesion depth and thermal signal intensity will be critical to understanding IR’s limitation as a measure of skin temperature. Ultimately, integrating IR with structural imaging modalities may enable a more comprehensive assessment of calcinosis by combining sensitivity to disease activity with precise anatomical localization.

## 5. Conclusions

This paper demonstrates the feasibility of using IR imaging to detect temperature variations associated with calcinosis in DM/JDM patients. With appropriate further validation and refinement, IR imaging may ultimately serve as a complementary tool in the clinical monitoring of DM-associated calcinosis, particularly for longitudinal assessment and screening purposes.

## Figures and Tables

**Table 1:** Patient cohort demographics.

Category	Subcategory	Cohort Value (n = 15)
Height, mean ± SD, cm		159.0 ± 14.6
Weight, mean ± SD, cm		67.2 ± 32.1
Female, n (%)		73% (11)
Ethnicity, n (%)	Hispanic or Latino	13% (2)
Race, n (%)		
	White or Caucasian	80% (12)
	African American or Black	13% (2)
	Asian or Asian American, Pacific Islander	0% (0)
	Mixed Race	7% (1)
JDM Diagnosis, n (%)		73% (11)
Age at imaging, mean ± SD, y		27.1 ± 16.1
Disease duration at time of imaging, mean ± SD, y		13.6 ± 14.3
Autoantibody status n (%)		
	Negative	30.8%
	p155 (TIF1)	23.1%
	Mi-2	23.7%
	NXP2	30.8%
	Ro	23.1%
CDASI Activity, mean (range) (0–100)		12 (2–39)
CDASI Damage, mean (range) (0–32)		12 (4–20)
CDASI Total Calcinosis score, mean (range) (0–15)		5 (2–11)
Physician Global Activity, mean ± SD (0–10 cm)		1.3 ± 1.2
Physician Global Calcinosis Severity (0–10 cm)		3.8 ± 2.1

Mixed race participant identified themself as White or Caucasian, African American or Black, and as Asian or Asian American, Pacific Islander. Abbreviations: SD, standard deviation; JDM, juvenile dermatomyositis; CDASI; Cutaneous Dermatomyositis Disease Area and Severity Index.

**Table 2:** Calcinosis distribution in 15 patients with adult and juvenile dermatomyositis, as determined by an infrared imaging algorithm.

Body Region	Average PCA ± SD (%)	Calcinosis-Positive Frequency
Right Upper Extremities	13.2 ± 4.2	4/15 (26.7%)
Left Upper Extremities	14.2 ± 5.2	6/15 (40.0%)
Abdomen	12.7 ± 8.9	5/15 (33.3%)
Back	8.6 ± 8.3	4/15 (26.7%)
Right Lower Extremities	11.4 ± 4.0	4/15 (26.7%)
Left Lower Extremities	12.1 ± 6.0	6/15 (40.0%)

Abbreviations: PCA, Percent Calcinosis Area; SD, standard deviation.

## Data Availability

The data presented in this study are available on request from the corresponding author due to privacy and ethical reasons.

## Abbreviations

The following abbreviations are used in this manuscript:

JDM	Juvenile Dermatomyositis
DM	Dermatomyositis
DM/JDM	Adult and Juvenile Dermatomyositis
NIH	National Institutes of Health
CT	Computed Tomography
IR	Infrared Imaging
MRI	Magnetic Resonance Imaging
CDASI	Cutaneous Dermatomyositis Disease Area and Severity Index
PCA	Percent Calcinosis Area
